# Marine Excitatory Amino Acids: Structure, Properties, Biosynthesis and Recent Approaches to Their Syntheses

**DOI:** 10.3390/molecules25133049

**Published:** 2020-07-03

**Authors:** Valentin A. Stonik, Inna V. Stonik

**Affiliations:** 1 G.B. Elyakov Pacific Institute of Bioorganic Chemistry, Far Eastern Branch, Russian Academy of Sciences, prospect 100 let Vladivostoku, 159, 690022 Vladivostok, Russia; stonik@piboc.dvo.ru; 2 National Scientific Center of Marine Biology, Far Eastern Branch, Russian Academy of Sciences, ul. Palchevskogo, 17, 690041 Vladivostok, Russia

**Keywords:** kainic acid, domoic acid, dysiherbaine, neodysiherbaine A

## Abstract

This review considers the results of recent studies on marine excitatory amino acids, including kainic acid, domoic acid, dysiherbaine, and neodysiherbaine A, known as potent agonists of one of subtypes of glutamate receptors, the so-called kainate receptors. Novel information, particularly concerning biosynthesis, environmental roles, biological action, and syntheses of these marine metabolites, obtained mainly in last 10–15 years, is summarized. The goal of the review was not only to discuss recently obtained data, but also to provide a brief introduction to the field of marine excitatory amino acid research.

## 1. Introduction

Throughout the 55-year history of the G.B. Elyakov Pacific Institute of Bioorganic Chemistry (PIBOC), Far Eastern Branch, Russian Academy of Science, a vast variety of new marine-derived natural compounds including different glycosides, polar steroids, alkaloids, lipids, and other metabolites have been isolated from echinoderms and sponges, ascidians, algae, fungi, and marine bacteria and thoroughly studied. Many of the discovered new natural compounds proved to have a protective action and were repeatedly mentioned in well-known series of review articles published in Natural Products Reports [1]. To protect themselves against predators and pathogens, marine organisms use a variety of structural groups of toxins. The latter are synthesized by lower plants, including microalgae [2,3,4], and then accumulated by some invertebrates that transfer them up to higher trophic levels. Marine excitatory amino acids (MEAAs) that belong to this category and play an important role in marine communities have found application in pharmacology. To date, there are only three known groups of marine-derived excitatory amino acids: (**1**) kainic acid and related compounds, (2) domoic acid and some its derivatives, (3) sponge-derived dysiherbaine and neodysiherbaine A, which specifically bind and activate the kainate-type of glutamate receptors (GluRs).

**Figure 1:**
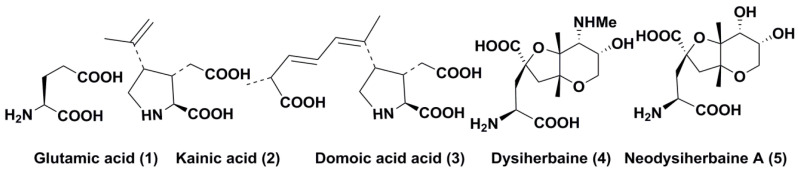
Chemical structures of glutamic acid (**1**) and marine excitatory acids (**2**–**5**).

MEAAs, including the main compounds shown in Figure 1, are a chemical group of particular research interest to us, although many of them were discovered long ago. This is explained by our studies on highly polar marine metabolites from northwestern Pacific invertebrates, including sponges, and marine bacteria and search for new excitatory compounds or their analogs. Another explanation is the long-term interest of one of the coauthors of the present review (S.I.) [5] in domoic acid (DA), and in DA-producers from the northern Pacific Ocean.

In general, these compounds are known as bioactive agents, particularly some of them as biotoxicants, contaminating marine waters. The application of these compounds in experimental pharmacology has expanded the knowledge about transmission of nerve impulses in higher animals. In fact, excitatory amino acids have a structure similarity with that of the principal excitatory neurotransmitter glutamic acid (**1**) and act as central nervous system (CNS) excitants. In the past decade, MEAAs, sometimes called kainoids, have become an important research field, with several discoveries made.

Despite the successes of the most recent studies on biosynthesis of these natural products, a number of questions about their appearance and disappearance in producers and consumers, transfer via food chains, action on own producers, and origin of some of them still remain unresolved.

The last review [6] considering excitatory amino acids was published more than 20 years ago. Herein, we discuss the recent results of studies on marine-derived natural products belonging to this group, including their discovery, structure, biosynthesis, recent approaches to syntheses, origin, biological action in producers and recipients, and environmental roles.

## 2. Kainic Acid and Related Metabolites

### 2.1. Discovery, Structure, and Some Properties

**Figure 2:**
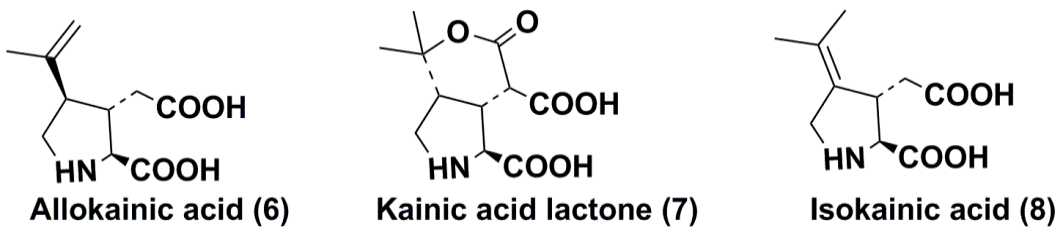
Some compounds closely related to kainic acid (**6**–**8**).

The key member of the kainoid family, kainic acid (**2**) [(–)α-kainic acid], sometimes referred to as digenic acid, was isolated by Japanese chemists in the 1950 s from aqueous extracts of the red macroalga Diginea simplex (Floridiophyceae, Ceramiales, Rhodomelaceae). Compound **2** in these extracts was used as a vermifuge in the traditional medicine of East Asian countries for over a thousand years [7]. In 1953, Murakami and co-authors [8] showed this acid to be the most active anthehelmintic agent from this alga and named it kainic acid after Kaininso, the Japanese name of this alga. The structures of this compound and its isomer, allo-kainic acid (**6**), were established by X-ray analysis of the zinc salt of kainic acid and, later of kainic and allokainic acids themselves [9,10]. Compound (**2**) was also detected in several other species of lower macrophytes such as the red alga Centroceras clavulatum [9] and others [11]. The Corsican moss Alsidium helminthochorton [12] contains kainic acid (**2**), allokainic acid (**6**), and α-kainic acid lactone (**7**) (Figure 2) along with a peptide of 37 amino acids including two kainic acid residues. A mutant strain of the alga Palmaria palmata was found to produce high levels of kainic acid [13]. Chemical transformation of **2** provided several additional compounds of this series such as isokainic acid (**8**) [12].

A new stage in the fundamental research of kainic acid came when its most interesting property, the ability to specifically activate a subgroup of glutamic acid receptors, was discovered in the early 1970s. Later, these receptors were named kainate receptors (KARs) [14,15,16]. Kainic acid is one of the best natural agonists of KARs. It causes the influx of cellular Ca^2+^ ions, production of reactive oxygen species, and mitochondrial dysfunctions that leads to neuronal apoptosis and necrosis. Hyperstimulation of KARs is involved in the pathogenesis of various neurodegenerative disorders such as epilepsy, Hantington’s chorea, and stroke. These properties of **2** and its application in experimental neurobiology and pharmacology have attracted much attention and stimulated development of different approaches to synthesize this excitatory acid and related compounds. In 1995, after the discovery of better anthelmintics than **2**, the production of this acid as anthehelmintic in Taiwan was stopped. However, application in experimental pharmacology continued to require increasing amounts of this compound, and it began to be produced not only from algae, but also by synthesis. In the early 2000s, its price increased more than 50 times, to $100 per 10 mg [17]. In 2012, Evens and Inglesby [18] noted that kainic acid had an estimated market value of 1 billion USA $ per annum. The price of synthetic (−)-α-kainic acid from Sigma-Aldrich in January 2012 was $750/50 mg and increased up to $1749/50 mg by May 2020. The deficit and the exceptionally high price of this product necessitated development of optimal schemes for its complete syntheses, the number of which has increased significantly in the recent decade.

### 2.2. Recent Syntheses

Thus, kainic acid raised great interest in synthetics due its activity, wide use in experimental pharmacology, and probability to synthesize analogs and derivatives which could be applicable in medicine to treat schizophrenia and other brain diseases. Taking into account that configurations of its three stereogenic centers are crucial in binding to receptors and functional activities of this compound, the stereoselective synthesis of **2** was directed first to the optically active form of **2**, identical to the natural product. Different synthetic strategies, based on C2–C3 or C3–C4 bond formations (the latter approach was used in majority of syntheses), C4–C5 bond formation and C-*N* bond formation pathways, as well as on the use of the existing pyrrolidine ring and cycloaddition reactions, were applied to synthesize this excitatory amino acid. As a result, about 40 total multi-step syntheses of **2** were reported in literature and reviewed in 2012 [19].

In our review, we discuss only some of syntheses reported later. Most of them are based on novel schemes. We omit listing all the stages of these syntheses here and, instead, pay attention to main ideas and results achieved in comparison with the already known approaches.

**Scheme 1:**
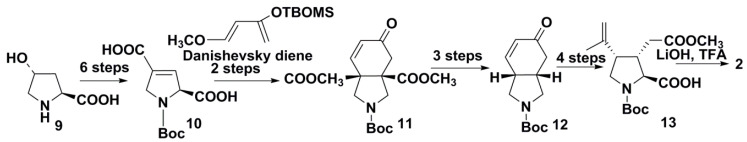
Synthesis of kainic acid by Diels-Alder reaction.

Poison et al. [20] elaborated an efficient synthesis of (–)-kainic acid (**2**) using just two of the above-mentioned approaches: application of a pyrrolidine precursor and cycloaddition. A high-pressure Diels-Alder cycloaddition of the obtained from 4-hydroxy-l-prolin (**9**) 3,4-unsaturated pyrrolidine derivative **10** with Danishefsky’s diene at high pressure (15 kbar) and room temperature gave bicyclic product **11**. In this case, high pressure provided the gain in reactivity and led, after some additional treatment and 82 h of exposure, to a 96% conversion into **11** that contained *N*-protected trisubstituted pyrrolidine cycle. The subsequent decarboxylation and transformation of its six-membered ring to isopropenyl and carboxymethyl groups gave **12** converted into target product via pyrrolidine derivative **13** with almost total stereocontrol and an approximately 10% yield (Scheme 1). At the same time, the key intermediate product, enone **11**, and related compounds were partly racemic in their first synthesis attempts. However, preliminary purification of the initial Danishevsky’s diene by rapid distillation and removal of trace triethylamine from it made it possible to synthesize **11** with excellent yield and high enantiomeric excess.

**Scheme 2:**
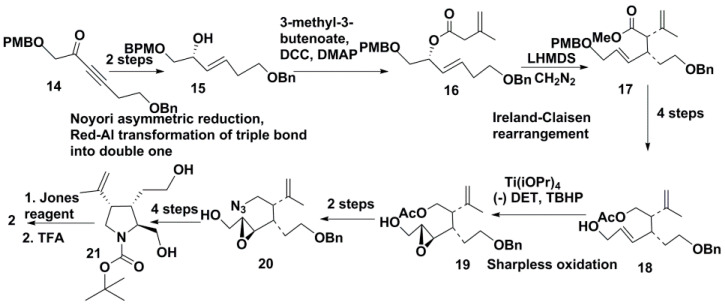
A shortcut scheme of synthesis of kainic acid via chirality transfer through the Ireland-Claisen rearrangement.

New approaches, based on the formation of C3–C4 bond and involving the Ireland-Claisen rearrangement of allylic esters, were used by Indian and Japanese groups. Reddy and Chandraseker [21] utilized this rearrangement along with the Sharpless asymmetric epoxidation. The first of these reactions was used to create C3 and C4 *cis* stereocenters, while the Sharpless oxidation allowed the designing of chirality at C2. Authors constructed the target compound 2 from ynone **14**. At the first stages, this compound was converted into alcohol **15** by Noyori reduction and Red-Al transformation of triple bond into double one. Reaction of **15** with 3-methyl-3-butenoate catalyzed by *N*,*N*′-dicyclohexylcarbodiimide (DCC) and 4-dimethylaminopyridine (DMAP) gave the allylic ester **16**, which was converted into **17** by the Ireland-Claisen rearrangement with lithium bis(trimethylsilyl)amide (LiHMDS) and trimethylsilyl chloride (TMSCl). The subsequent four steps of transformations led to allyl alcohol **18** with removal of p-methoxybenzyloxy (PMBO) protective group. The obtained **18** was epoxidated by asymmetric Sharpless epoxidation using (–)-diethyltratrate (DET), titanium isopropoxide Ti(iOPr)_4_, and *tert*-butyl hydroperoxide (TBHP) to give **19**. Further conversion provided azide **20**. Reduction of **20** allowed the formation of pyrrolidine ring, and then NH in this ring was protected by reaction with *tert*-butyloxycarbonyl anhydryde ((tBoc)_2_O) to obtain **21** in several steps. Removal of the protective group and oxidation with Jones reagent (CrO_3_ in aqueous sulfuric acid) led to kainic acid (**2**) (Scheme 2). Thus, a new strategy for constructing the core system of **2** was realized in this synthesis.

**Scheme 3:**
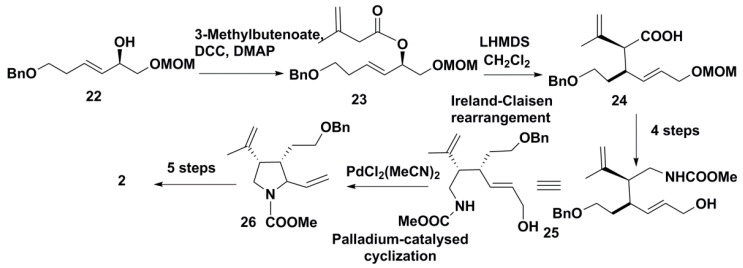
Synthesis of kainic acid using the Ireland-Claisen rearrangement and palladium catalyzed formation of pyrrolidine ring.

A similar strategy, also based on Ireland-Claisen rearrangement, was proposed by Japanese scientists as a unified approach to synthesize not only kainic acid proper, but also bioactive 4-substituted kainoids [22]. A source compound **22** was obtained from l-tartaric acid through four steps. Condensation of 22 with 3-methyl-3-butenoic acid gave ester **23** which, through the Claisen-Ireland rearrangement with LHMDS in the presence of TMSCl, was converted with high diastereoselectivity into carbonic acid **24**. The subsequent reduction of a carboxy group and other transformations gave aminomethyl derivative **25**. The resulting product was cyclized exceptionally easily through the palladium-mediated pyrrolidine-ring formation into the product **26** that had the required stereochemistry for transformation into kainic acid or its derivatives. After protection of the isopropenyl group, the corresponding conversion into **2** was achieved by oxidative cleavage of the vinyl group by ozonolysis followed by the Jones oxidation (Scheme 3).

**Scheme 4:**
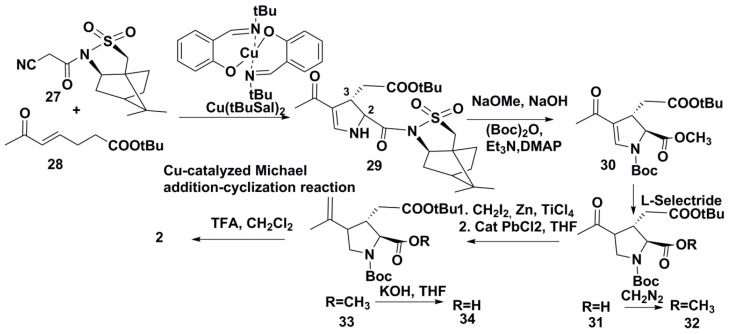
Short total synthesis of (–)-kainic acid.

The advantage of the recent synthesis of kainic acid, developed by Japanese scientists [23], unlike most other multi-stage syntheses, consisted in only nine stages. This short total synthesis was carried out on the basis of the Cu-catalyzed Michael addition-cyclization reaction between the bearing chiral auxiliary of camphorsultam-type isonitrile **27** and ester of unsaturated ketoacid **28** (in mixture with an isomeric ester) with Cu(t-ButSal)_2_ as catalyst (Scheme 4). The additional ester was isomerized into **28** in the conditions of this reaction. The treatment of the obtained adduct **29** by sodium methoxide with the loss of chiral substituent, followed by protection with (Boc)_2_O in the presence of triethylamine and DMAP, gave unsaturated pyrrolidine **30**. The alkaline hydrolysis of the amide group at the position 2 and the selective reduction of a double bond by boron-organic reagent l-selectride led to the protected analog of kainic acid **31** which was converted into the target compound via intermediates **32**–**34**. This synthesis was carried out with a 16.8% overall yield on chiral isonitrile and was conducted on a 300 mg scale, although usually syntheses of **2** had given smaller amounts of this product.

**Scheme 5:**
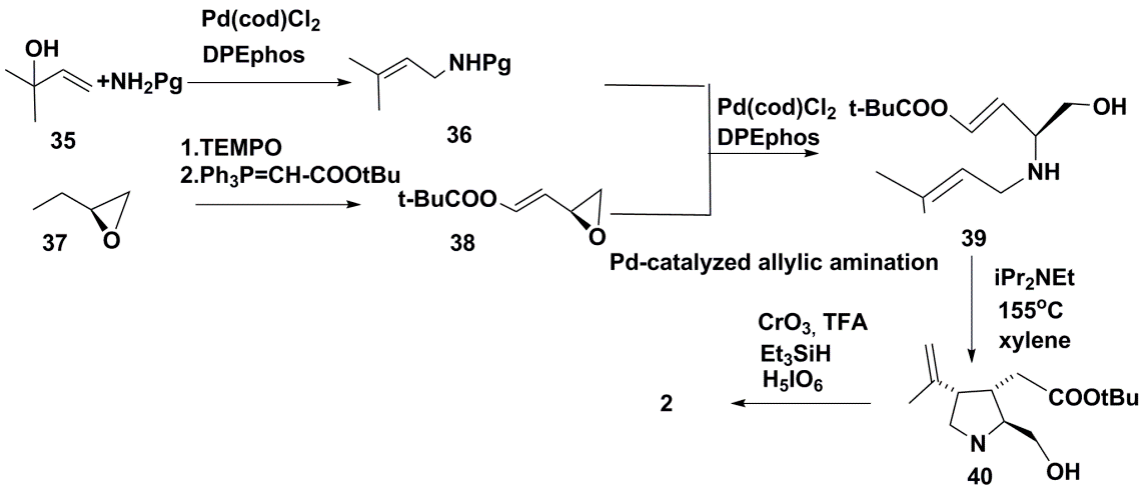
Synthesis of kainic acid using the Pd-catalyzed direct allylic amination.

A gram-scale synthesis of (–)-kainic acid through six steps and with a 34% overall yield was elaborated using the Pt-catalyzed direct allylic amination [24]. This amination was catalyzed by the combination of dichloro(1,5-cyclooctadiene) platinum (Pt(cod)Cl_2_) with bis[2-di-phenylphosphino) ether (PPEphos). Such new approach allows catalyzing the direct introduction of amino group into allylic alcohols. The chosen scheme provided the minimum number of stages in this synthesis (Scheme 5). Amination of **35** under microwave heating conditions gave monoallylamine **36**. As a protective group (Pg), authors preferred to use 2,4-dimethoxybenzyl (DMB) or p-methoxybenzyl (PMB) with almost no loss of enantiopurity. The unsaturated ester **38** was obtained by one-pot oxidation of epoxide **37** with 2,2,6,6-tetramethyl-1-piperidinyloxy (TEMPO) followed by the Wittig reaction. It was found that the transformation of **36** and **38** into diallylamine derivative **39** at a higher concentration inhibited the epimerization, and the desired product was obtained in 95% yield and 98% ee (enantiomeric excess). The following stage of heating in a sealed tube with xylenes and a catalytic amount of *i*Pr_2_N-Et gave pyrrolidine **40** in 75% yield and 14:1 diastereoselectivity. The Jones oxidation with application of H_5_IO_6_ as terminal oxidant gave satisfactory results at conversion of **40** into **2** (Scheme 5). Finally, 1.11 g pure kainic acid was obtained by this method in 34% overall yield. Taking into account its yield and the minimal number of steps, this proved to be the best chemical scheme elaborated to synthesize **2**.

**Scheme 6:**
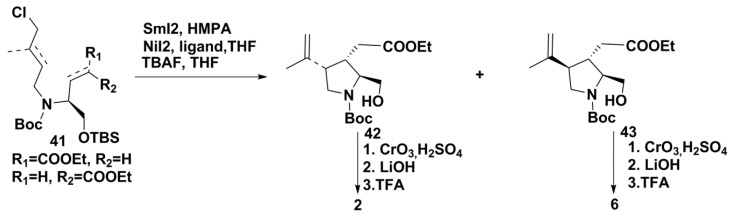
Syntheses of kainic and *allo*-kainic acids using SmI_2_-induced cyclization.

Kainic and allo-kainic acids (**2** and **3**, respectively) were also synthesized using SmI_2_, a unique single-electron reducing agent that allows the induction of reductive coupling with the formation of C–C bond under mild conditions [25]. The key initial products for this new synthesis were obtained from D-serine methyl ester hydrochloride using the known procedure and a mixture of E- and Z-isomers of α,β-unsaturated esters, designated as E-14 and Z-14, with a general formula of **41**. The cyclization of **41** gave derivatives of kainic and/or allo-kainic acids (**42** and **43**) with different yields depending on ratio of the reagents (SmI_2_, hexamethylphosphoramide (HMPA), NiI_2_ and H_2_O) and additional ligands such as ethylenediamine, 2,2′-bipyridine, 2,2′-bipyridylamine, triphenylphosphine, etc. Pure compounds were isolated in optimal conditions of this reaction for each of them followed by separation and purification of the products through HPLC. The obtained compounds were converted into kainic acid **2** or allo-kainic acid **3** through a 3-step transformation by conversion ester groups into acids and deprotection of nitrogen in pyrrolidine ring (Scheme 6).

The formal total synthesis of (–)-kainic acid, recently described by Chinese chemists [26], was based on a new approach to synthesis of the same important intermediate, which was already used for synthesizing kainic acid by different authors [19,20].

In general, there have been several syntheses of kainic acid published in recent years, which confirms the constant attention to this excitatory amino acid and the rapid progress of organic synthesis. These syntheses are shorter and can provide better production of kainic acid as a pharmacological probe for application in experimental pharmacology, although the costs and availability of the synthetic precursors and reagents used were not compared with previously known syntheses. All the studies have discovered a large number of kainoids, a total of 70 [26], including those obtained through recent chemical syntheses.

### 2.3. Biosynthesis and Application of Kainoids

**Scheme 7:**
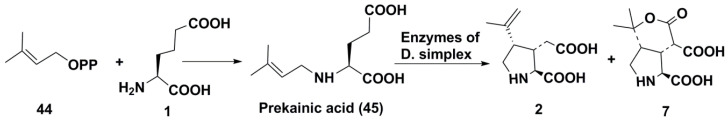
Biosynthesis of kainic acid.

Recently, Moore’s team from the USA studied the biosynthesis of two related excitatory amino acids: domoic acid from diatoms and kainic acid from red macrophytes. After elucidating the origin of kainic acid **2**, they performed the whole genome sequencing of a kainic acid producer, *Digenea simplex*, to identify the gene clusters involved in the biosynthesis of **2** [27]. They used the new single-molecule, long-read sequencing platforms such as Oxford Nanopore Technologies. As a result, the long reads that included genes encoding the kainic acid biosynthesis were sequenced; however, due to the presence of impurity of microbial DNA, they were not assembled into the whole genome of this alga. After the analysis of *D. simpex* on a higher-throughput platform, Prometh ION, 47 Gp of sequence was generated, with the longest read being 1.2 Mb. A series of *Digenea simplex* kainic acid biosynthesis (Dskab) genes were uncovered. They included genes of annotated *N*-prenyltransferase (DsKabA), α-ketoglutarate-dependent dioxygenase (DskabC), and several retrotransposable elements such as integrase, reverse transcriptases, and RNA H domains. Both sequences, DsKabA and DskabC, were successfully expressed in *Echerichia coli* and purified. Incubation of recombinant DskabA with l-glutamate **1** and dimethylallylpyrophosphate **44** gave *N*-dimethylallyl-l-glutamic acid **45** (the so-called prekainic acid). This compound was converted into kainic acid in an aqueous extract from *D. simplex.* Another enzyme of kainic acid biosynthesis, the so-called DsKabC, was incubated with prekainic acid, α-keto-glutarate, l-ascorbate, and Fe^2+^, and transformed prekainic acid into kainic acid (**2**) and kainic acid lactone (**7**) (Scheme 7). The main product was confirmed to be kainic acid using isolation from the culture medium and NMR elucidation. The obtained kainic acid lactone **7** has been shown to be antagonist of iGluR in contrast to kainic acid proper.

To show the power of both chemical synthesis and biocatalysis, prekainic acid was synthesized through the reductive amination of l-glutamate with 3-methyl-2-butenal, by the method reminiscent of the cobalt-mediated strategy of Baldwin et al. [28]. For the next step, DsKabC was used without additional purification, but in a medium with E. coli expressing this enzyme. The employment of this combined approach and the purification procedure using activated carbon followed by preparative reverse phase HPLC provided 1.1 g of kainic acid with a 32% overall yield and 95% purity. It proved to be better compared to any chemical synthesis. Thus, this work has not only discovered enzymes of biosynthesis of kainic acid, but also developed a new method combining chemical and enzymatic transformations to obtain **2** for pharmacological research.

Analyses of genomes of other red algae revealed similar genes. For example, genes named as PpkabA and PpkabC were identified in the red alga Palmara palmata, the second known producer of **2**. In comparison with Digenea simplix, the kab genes in the edible P. palmata were tightly clustered, and the retrotransposable elements were absent in the intergenic region. The corresponding kab genes were also found in four other red algae species [27].

As it is well known, **2** has found a wide range of applications to model epilepsy, to determine molecular events indicating human neurodegenerative disorders, and to evaluate efficiency of various therapeutic interventions on laboratory animals with simulated diseases [29,30,31].

The specific interaction of kainic acid with glutamic acid receptors (GARs) in CNS was studied. Most of ionotropic kainate receptors have ligand-binding domains which form a clamshell-like structure consisting of lobes. Kainic acid, like glutamic acid, is bound between these lobes, but their influence on activities of these receptors remains to be explained [32]. As a neurotransmitter, kainic acid acts also on another class of receptors, metabotropic glutamate receptors (mGluRs), which respond slower than iGluRs, but their interaction with **2** shows an effect on the learning and memory functions.

The biological role of kainic acid in its producers was more poorly studied than distribution, biological activities, and biosynthesis. Using rabbit polyclonal antibodies for kainic acid, which did not cross-react with other amino acids including glutamate, Japanese scientists immunochemically localized this toxin in the fine cylindrical thallus of D. simpex [33]. They concluded that the presence of kainic acid on the surface of the alga can be related to its role as a protection against grazers.

Little is currently known about the functions of algal glutamate receptors-like compounds or the probable influence of kainic acid on these receptors in algae. However, the highest variability in glutamate receptors sequences was recorded from algae [34]. These receptors are associated with many plant-specific physiological functions, such as sperm signaling in moss, pollen tube growth, root meristem proliferation, and innate immune and wound responses. It should be taken into account that the main physiological roles and modes of action of plant GluRs, in contrast to those in animals, are performed in peripheral, non-neuronal tissues [35].

**Figure 3:**
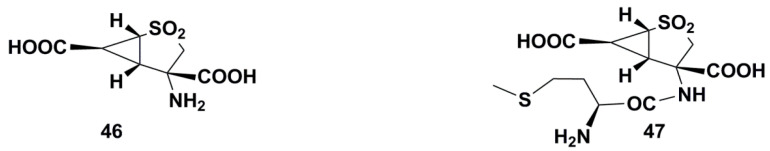
Structures of synthetic derivatives of glutamic acid (**46**,**47**).

Further studies on some synthetic derivatives of kainic acid and analogs (Figure 3) are aimed, first, at discovering the earlier unknown events in the brain depending on kainate receptors. Second, analogous compounds can be used to create novel drugs. Some of these compounds have reached clinical trials. For example, the compound LY404039 (**46**) is a selective agonist for the metabotropic glutamate 2/3 (MGlu2/3) receptor. This preparation, when dosed as prodrug (**47**) (LY2140023), showed efficacy in schizophrenia. A series of similar compounds was studied as potential psychiatric medicines by such pharmaceutical companies as Lilly and Taisho [36].

## 3. Domoic Acid and Related Compounds

### 3.1. Discovery, Structure, and Some Properties

Domoic acid (DA) **3** was first found and isolated from the red alga *Chondria armata* by Japanese scientists in the 1960s [37]. After three years of studies, its structure was determined and then refined through organic synthesis. Like kainic acid, **3** is a derivative of dicarboxylated pyrrolidine containing glutamate moiety, but in its another moiety, domoic acid bears octadienoic substituent derived from a monoterpenoid acid (Figure 1). Closely related metabolites, isodomoic acids **48**–**55**, were identified for more than 25 subsequent years in the red alga *C. armata* and contaminated mussels [38,39,40], as reviewed in [41,42]. All these metabolites, including isodomoic acids A-H along with domoilactones and some related compounds, also contain a glutamic acid residue and should be considered as effectors of GARs. However, as a rule, their effect is much weaker (Table 1). The number of known analogues of DA has increased, particularly in recent years [43].

**Figure 4:**
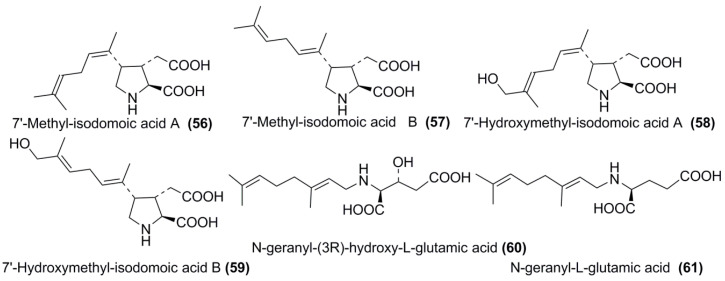
Dainic acids (**56**–**59**) and related metabolites (**60**,**61**) from *C. armata*.

Several compounds related to domoic acid (dainic acids) have recently been described and named as 7′-methyl-isodomoic acid A (**56**), 7′-methyl-isodomoic acid B (**57**), 7′-hydroxymethyl-isodomoic A (**58**), and 7′-hydroxymethyl-isodomoic acid B (**59**). Closely related minor metabolites **60**, **61**, which do not contain pyrrolidine ring, have also been isolated from the same alga *C. armata* (Figure 4) [43].

The main turn in the fate of domoic acid and related compounds occurred after the discovery that these metabolites are produced not only by several species of red algae, but also by planktonic diatoms [43]. As a result of subsequent transfer of domoic acid (DA) (**3**) up the food chain from microalgae into edible mollusks [44,45,46], it may cause the so-called Amnesic Shellfish Poisoning (ASP) in humans that consume edible mollusks contaminated by DA [47,48,49]. The first case of ASP was described in 1987 from Canada, where 107 people, who had consumed cultivated mussel *Mytilus edulis*, showed symptoms of this disease and three of them died [45]. The diatom *Pseudo-nitzshia multiseries* was identified as a phytoplanktonic producer of DA, which also produces isodomoic acids E and F. Since 1987, it was found that this toxin and related compounds are responsible not only for human poisoning, but also for numerous cases of poisoning and mortality of birds and marine mammals [46,47,48,49].

Symptoms of ASP in humans include gastrointestinal alterations (abdominal cramp, nausea, vomiting), and/or neurological disorders (dizziness, short-term memory loss, seizure, epilepsy or coma in the acute cases) [47]. DA binds to glutamate receptors in the central nervous system (CNS) and myocardium [50] causing overexcitation and, as a consequence, neuro-excitatory behavior in humans, marine mammals, and fish [51,52,53,54,55]. The binding of DA to kainate or AMPA (α-amino-3-hydroxy-5-methyl-4-isoxazolepropionic acid) receptors, subclasses of glutamate receptors, results in the intracellular accumulation of Ca^2+^ [47,55]. In contrast to glutamic acid, DA causes long-lasting depolarization, neuronal swelling induced by intraneuronal accumulation of excess Ca^2+^, production of reactive oxygen species, DNA and mitochondrial damages, energy depletion, and cell death [55,56]. Along with the CNS, the heart is often also considered as a potential target site for adverse effects of DA. DA-induced cardiotoxicity has been confirmed not only in people suffering from ASP, but also in sea lions and zebrafish that died as a result of DA-poisoning [51,52].

Isodomoic acids (iso-DAs), which have an insecticidal effect against the American cockroach *Periplaneta americana* [38], were also isolated from marine diatoms including *P. australis*, *P. seriata*, and *Nitzschia navis-varingica* [57]. Some diatom species (e.g., *Nitzschia navis-varingica*) produce isodomoic acids A and B as the major toxin components [49]. However, iso-DAs are often present in the environment at lower concentration than DA [57]. The affinity of iso-DAs to the glutamate receptors is lower than that of DA (Table 1) [58]. Therefore, parent toxin is a major threat for humans and animals, in contrast to iso-DAs [59].

Thus, a series of DA isomers have been identified, while data on occurrence of DA and epidomoic acid (referred to as total DAs) and their toxicity have been used to establish the current regulation limits for DA of ≤20 mg∙kg^−1^ [54]. The discovery that exposure to low levels of DA leads to long-lasting neurological effects in mammalian species [53] may suggest the need to reduce its permissible level in tissues of consumed finfish and shellfish [57].

### 3.2. Synthesis

**Scheme 8:**
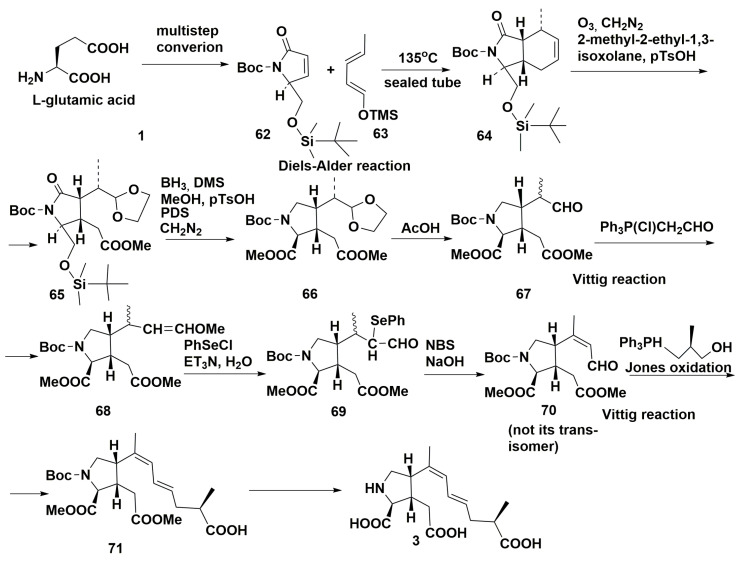
Synthesis of domoic acid.

Synthesis of the domoic acid **3** and correction of the earlier proposed geometry of the double bonds in its side chain have been carried out [60]. The bicyclic core was constructed from the compound **62**, obtained by multistep synthesis from **1** and diene **63** using the thermic Diels-Alder reaction to give bicyclic adduct **64** with *cis*-fused ring junction. This adduct was ozonized, treated with diazomethane and 2-methyl-2-ethyl-1,3-dioxolane in the presence of p-TsOH and yielded 1,3-dioxolane **65**. The employment of borane-dimethylsulfide complex reduced the carbonyl group. Deprotection of the silyl ester and subsequent oxidation of intermediate diol with pyridinium dichromate (PDC) provided the diester **66**. Selective removal of ketal group by 60% AcOH gave the aldehyde **67**. The Wittig reaction led to **68**, the subsequent reaction with PhSeCl to the selenium-containing aldehyde **69**. The key conversions of this synthesis consisted in the obtaining of *trans*- and *cis*-enal systems from **69**. For example, the treatment with *N*-bromosuccinimide (NBS) in tetrahydrofuran and then with NaOAc led to *cis*-enal **70** as a major product. Transformation of this particular compound, but not its trans-isomer by the Wittig reaction followed by the Jones oxidation gave the product **71**. The subsequent removal of protective groups converted it into domoic acid **3**, thus confirming the *E,Z,R*-configurations in the side chain of this toxin (Scheme 8).

### 3.3. Biosynthesis, Producers, Biological Action, and Environmental Role

Several attempts were made to study the process of biosynthesis of this toxin in order to understand how and why environmental factors trigger the biosynthesis leading to high levels of DA accumulated in diatoms. Using feeding experiments, Savage et al. [61] established that DA arises as a result of condensation of geranyl diphosphate (**72**) with glutamate. Labeled by a stable isotope (deuterium), geranyl diphosphate was incorporated from culture medium into DA that was confirmed by gas chromatography–mass spectrometry (GC-MS). It was suggested that the condensation of geranyl diphosphate with amino group glutamic acid occurred via nucleophilic substitution of the diphosphate group with the amino group of glutamic acid (**1**).

**Scheme 9:**
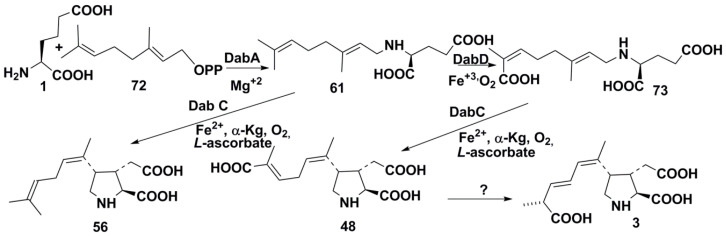
Biosynthesis of domoic acid.

A recent publication in Science [62] provided new important data concerning the DA biosynthesis. It was shown that these genetically programmed biosynthetic pathways are encoded by a four-gene cluster which is involved in this biosynthesis and exists in genomes of toxic microalgae belonging to the genus *Pseudo-nitzschia*. In the conditions of phosphate limitation and elevated CO_2_ level, probably characteristic of the final stage of microalgal bloom, *Pseudo-nitzschia* spp. express a series of enzymes involved in construction of the pyrrolidine skeleton system of DA. The up-regulation of CYP450 gene, encoding an enzyme which is responsible for the formation of 7′-carboxylic acid from geranyl diphosphate **72** at its reaction with glutamic acid (**1**), was revealed. It was shown that this gene is clustered with several other genes of DA biosynthesis (the so-called dab genes). The DA biosynthetic gene cluster was established to consist of terpene cyclase (dabA), hypothetical protein Hypo (dabB), dioxygenase (dabC), CYP450 (dabD), and probably other genes. The expression of recombinant dabA without *N*-terminal transit peptide in *E. coli* and its use as a catalyst in the reaction between **72** and glutamic acid **1** confirmed this enzyme to catalyze *N*-geranylation of l-glutamic acid in Mg^2+^-dependent manner. This is the first and key stage in the DA biosynthesis, which yields *N*-geranyl-l-glutamic acid **61**, as was earlier suggested by Savage et al. [61]. The use of recombinant dabC in the presence of Fe^3+^, oxygen, and l-ascorbic acid, and α-ketoglutarate-dependent dioxygenase, or both dabC and dabD in one put, gave one of so-called dainic acids (**56**) with a non-oxidized geranyl side chain. When the primary product of this biosynthesis **61** was incubated with *Saccharamyses cerevisae* microsomes, which expressed the transmembrane dabD, *N*-geranyl-l-glutamic acid in the presence of Fe^3+^ and oxygen was converted into small, but reproductible amounts of 7′-carboxy-*N*-geranyl-l-glutamic acid (**73**) and its 7′-hydroxy analog. Both products were identified by being compared with synthetic fractions. The product **73,** in the same conditions as were used for the transformation of **61** into **56**, was converted into an isomer of domoic acid **48**. However, no isomerase activity necessary for the conversion of **48** into the end product of this biosynthesis was found (Scheme 9). All the above results show that this biosynthesis pathway begins with the dab A-catalyzed geranylation of l-glutamic acid in chloroplasts. Therefore, the presence of dab genes is a character that allows the recognition of toxic strains of this genus. It can help identify environmental conditions, which may potentially cause neurotoxicity in microalgae and in their consumers such as mollusks and diatom-feeding invertebrates.

Thus, it has been confirmed that DA, as a potent excitatory amino acid, is produced primarily by *Pseudo-nitzschia* and *Nitzschia* diatoms distributed across the world and is naturally accumulated in filter-feeding marine organisms. Although many *Pseudo-nitzschia* species have the potential ability to produce this neurotoxin [49], *P. multiseries*, *P. seriata*, and *P. australis* were established as the most toxic species [57].

There have been numerous bloom events caused by highly toxic DA-producing microalgae along the USA and Canadian coasts over the past 15 years. In many cases, it led to mass poisoning of marine mammals and birds [48]. For instance, in the spring of 2015, an unusually intense and long-lasting toxigenic bloom of *Pseudo-nitzschia* microalgae associated with an abnormal warming of water, was observed in a vast area from California to Alaska. Besides the mass poisoning of marine animals, it caused significant economic losses due to the closure of farms that cultivated bivalves and crabs [63]. Thus, DA exposure has become more widespread due to the higher intensity of toxigenic *Pseudo-nitzschia* blooms and related consumption of DA-contaminated mollusks.

A suggestion can be made that the isomerase activity necessary for the transformation of less active biosynthetic precursors into DA may be present not only in these microalgae but also in consumers, or in symbionts and/or epiphytic microorganisms. It is possible that bacteria play an important role in accumulation of DA in microalgae, but the details of this are still elusive [47]. It was established that axenic diatom cultures produce less DA than xenic ones and that bacteria can enhance DA production [64]. It was shown, for example, that after the addition of the gamma-proteobacterium *Alteromonas macleodii*, isolated from the Russian clone of *P. multiseries*, to an axenic culture of another clone of this species, the amount of DA produced by the latter significantly increased [65]. The stimulating effect of bacteria on the DA production has also been found for another known DA-producer, *Nitzschia* sp. [66]. However, mechanisms of bacteria’s influence on intracellular DA levels in microalgae remain insufficiently studied [49].

There are a few other hypotheses that discuss environmental factors influencing toxicity of diatoms. It was suggested that DA production may arise as a defense against grazing. However, the effect of predatory copepods on toxicity of microalgae has not been definitively confirmed [67]. The hypothesis that DA may be involved in a high-affinity iron uptake system seems likely [68]. Trick et al. [69] demonstrated that the addition of iron stimulated the growth of toxigenic *Pseudo-nitzschia* spp., providing a competitive advantage over other phytoplankton. Each of these hypotheses requires further validation.

## 4. Dysiherbaine and Related Compounds

### 4.1. Discovery and Structures

**Figure 5:**
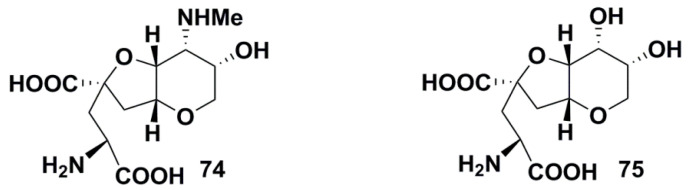
Structures of dysiherbaine (**74**) and neodysiherbaine (**75**).

In 1997, Japanese scientists described a new potent marine excitatory amino acid **74** [70], isolated through homogenization of the sponge *Dyidea herbacea* in water, centrifugation of the extract, and precipitation of high-molecular-weight compounds with 2-propanol. Further purification of water-soluble materials using several types of column chromatography yielded this toxin known as disyherbaine (Figure 5). Its structure was identified as a novel diamino dicarboxylic acid using NMR, FABMS, ISIMS, and other methods of molecular structure analysis. Structurally, the acid contains the bicyclic core consisting of *cis*-fused tetrahydropyran and tetrahydrofuran rings with a glutamic acid fragment attached to the tetrahydrofuran moiety. Dysiherbaine core contains four contiguous stereogenic centers with an additional quaternary stereocenter in the tetrahydrofuran moiety at the site of amino acid section attachment. This excitatory acid stimulates binding of kainic acid and 1-amino-3-hydroxy-5-methyl-4-isooxazolepropionic acid, but not *N*-methyl-D-aspartic acid to the rat brain synaptic membranes, suggesting that **74** is an agonist of KARs in CNS.

Radioligand binding assay showed that dysiherbaine binds kainic acid receptors at significantly lesser concentrations than other agonists of KARs. Intraperitoneal injection into mice (20 µg/kg) caused the same behavior as that observed after injection of domoic acid. In general, dysiherbaine exhibits the most potent epileptogenic activity among so far known amino acids. ED_50_ for substitution of kainic acid, labeled by tritium in kainate receptors by dysiherbaine is 0.00074 µM. This excitatory amino acid is useful for evaluating the physiological roles of KARs in the central nervous system [71].

The above-mentioned findings explained the increased attention to synthesis **74** and related compounds. Another excitatory amino acid, neodysiherbaine A **75** (Figure 5), was isolated by a bioassay-guided chromatographic procedure as a minor constituent of the aqueous extract from the same sponge species. Its structure was deduced by NMR and MS methods and unambiguously confirmed by the total synthesis. After HPLC purification, Japanese scientists obtained as small amount of **75** as 0.26 mg [72]. Neodysiherbaine A is also potent agonist of KARs which induces characteristic epilepsy-like seizures in mice. Like dysiherbaine, it activates neuronal glutamate receptors with a considerable preference toward kainic acid receptors. However, neodysiherbaine is less active in comparison with dysiherbaine: the same activity associated with crowding out kainic acid from kainate receptors is induced by 0.052 µM of neodysiherbaine [72].

After the discovery of dysiherbaine, it was established that various fragments of its structure play different roles in interaction with receptors. The glutamate section of this molecule is responsible for binding to a receptor, while structural and stereochemical features in the tetrahydropyran ring determine the profile of its activity, in particular, agonistic or antagonistic properties [70,71].

### 4.2. Some Recent Approaches to Syntheses

These compounds represent a new structural group of amino acids containing a unique hexahydrofuro[3,3–b]pyrane core system. It was shown that **74** and **75**, as well as many their synthetic analogs, exhibit preferential binding to GluK1 kainate receptors and almost no activity towards AMPA receptors. In total more 35 syntheses of dysiherbaine and neodysiherbaine were reported. Most of them were reviewed in 2013 by Cachet and Poree [73]. In our review, we will only consider several additional syntheses which were developed later and published in 2013–2018.

Taking into account that several syntheses of dysiherbaine, reported earlier, were based on a known intermediate, obtained through 13 to 16 steps, a formal synthesis of **74** was carried out [74]. In this synthesis, D-mannitol diacetonide, an available initial compound, was converted into the same compound by a shorter synthetic pathway.

**Scheme 10:**
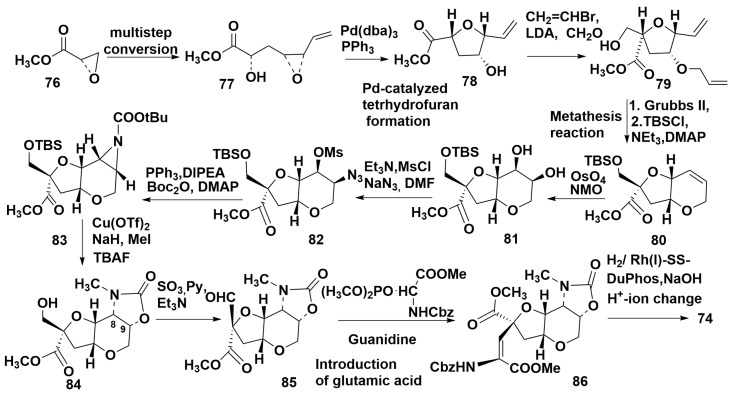
Enanthioselective synthesis of (–)-dysiherbaine.

Gilbeston and co-authors [75] from the University of Houston, USA, proposed a new variant of enantioselective synthesis of **74** which differed in a later design of functionalities in the tetrahydropyran ring than in other known pathways. The scheme of this synthesis suggested, first, the creation of tetrahydrofuran derivative and then the formation of tetrahydropyran ring with the use of metathesis reaction. The following introduction of the necessary substituents and, finally, the attachment of glutamic acid moiety to the furan fragment completed the synthesis. The furan fragment of **74** was constructed from methylglycidate **76**. This compound was transformed by a series of reactions into α,β-unsaturated epoxide **77**. The palladium catalyzed opening of this epoxide yielded the furan derivative **78**. Then **78** was converted by allylation of free alcohol with allyl bromide and silver oxide to provide the diene metathesis substrate **79**, in which oxymethyl group was introduced by reaction with LDA and formaldehyde. The derivative **79** was obtained as a mixture of stereoisomers and one of them, suitable for further transformations, was isolated by column chromatography. Metathesis of **79** with protection of hydroxy group gave **80**, containing the bicyclic core system of dysiherbaine. The oxidation of the double bond in **80** with OSO_4_ and *N*-oxide of methylmorpholine (MMO) led to diol **81**. On the following stages of introduction of mesyl and azide groups in the pyrane cycle was carried out to obtain **82**. The treatment of **82** with PPh3 and *N*,*N*-diisopropylethylamine (DIPEA) successfully provided aziridine **83**, protected with Boc. The consequent reaction with copper (II) triflate, methylation, and deprotection of oxymethyl group provided the rearranged production of **84**. Further steps were the transformation into aldehyde **85**, introduction of glutamic acid residue to obtain **86**. Asymmetric hydrogenation with rhodium DuPhos (DuPhos is a class of organophosphorus compounds that are used as ligands for asymmetric syntheses), followed by deprotection of amino group and alkaline hydrolysis gave the target compound **74** (Scheme 10).

**Scheme 11:**
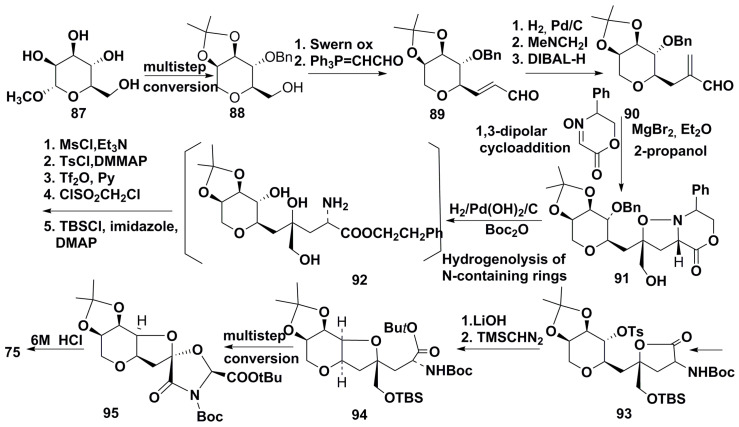
Synthesis of neodysiherbaine A from methyl-α-*D*-mannopyranoside.

A total synthesis of neodysiherbaine A (**75**) based on 1,3-dipolar cycloaddition of a chiral nitrone to sugar-derived product, containing tetrahydropyran moiety, has also been reported recently [76]. The pyran allylic alcohol was obtained from methyl-α-*D*-mannopyranoside (**87**) through conversion into derivative **88** by the previously known multistep procedure, and, after the Swern oxidation and Wittig reaction, gave **89**. The further hydrogenation and Mannich reaction with Eschenmoser’s salt yielded aldehyde, which, after DIBAL-H reduction, gave the compound **90**. The bipolar addition of a nitrone, catalyzed by etherate of MgBr_2_, led to **91**. This cycloaddition constructed the C2 and C4 asymmetric centers in a single step. Further transformations resulting in reductive cleavage of O-benzyl, *N*−O, and *N*-benzyl bond, which occurred simultaneously, led to intermediate **92** which was converted by multistep transformations into another key product (**93**). The intramolecular SN2 reaction in **93** led to the compound **94** containing the required configurations of all six stereogenic centers of neodysiherbaine A. The multistep substitution and removal of protective groups and formation of tricyclic intermediate **95**, followed by hydrolysis with 6M HCl, completed this synthesis (Scheme 11).

### 4.3. Probable Origin in Sponges

The cellular origin of **74** and **75** has remained a mystery for a long time, since excitatory acids are usually present in plants or microorganisms, but not in animals such as sponges. In 2008, using dysiherbaine antibodies, Japanese scientists from Kitasato University [77] have found dysiherbaine-to show immunoreactivity in spherical cells harbored in mesohyl of the sponge *Lendenfeldia chondrodes*. These spherical cells were identified as *Synechocystis* cyanobacteria by a combination of ribosomal RNA gene sequencing and cell morphology analysis. Therefore, these excitatory amino acids are probably formed in symbiotic cyanobacteria inhabiting some sponges.

## 5. Conclusions

Excitatory amino acids, found in marine organisms a few dozen years ago, continue to attract increasing attention by their unusual properties. Some of them, such as domoic acid and its analogues, are potent excitotoxicants harmful for humans and different marine mammals and birds. These substances may cause significant damage to both natural ecosystems and aquaculture farms. Kainic acid and some similar compounds, being specific agonists of kainate receptors, are used in experimental pharmacology to model epilepsy and other neurodegenerative diseases in animals. Application of these compounds have become an important approach to understanding of the biological roles, classification and modes of action of synaptic receptors. Other excitatory amino acids isolated from some sponges exert even more potent action on neurons of animals and humans than kainic and domoic acids do. The promising pharmacological properties of marine excitatory amino acids were an incentive for developing numerous syntheses of these natural products and their analogs.

The most interesting results of very recent research concerns the biosynthesis of kainic acid and chemico-enzymatic procedure, providing a more efficient obtaining of this compound than chemical syntheses. Another important discovery is the decoding of biosynthesis processes leading to domoic acid and isolation of a number of new natural derivatives of this excitant, so-called dainic acids. In the recent decade, the chiral organic synthesis allowed obtaining of kainic acid by short and effective pathways using different synthetic strategies. New hypotheses have been proposed that explain the biological origin of dysiherbaine and neodysiherbaine A in sponges from symbiotic cyanobacteria and the biological significance of kainic acid in algae producing this excitatory amino acid for their defense against grazers.

## Figures and Tables

**Table 1:**
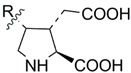
Metabolites related to domoic acid.

Compound	R (Side Chain)=	Name	KARs, μM	References
3	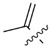	Domoic acid	2.2	[38,44,45]
48	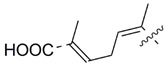	Isodomoic acid A	4.4	[39,42]
49	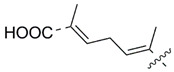	Isodomoic acid B	4990	[39,42]
50	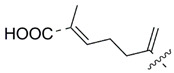	Isodomoic acid C	171	[39,42]
51	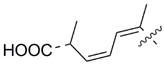	Isodomoic acid D	600	[40,41]
52	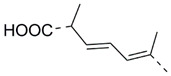	Isodomoic acid E	600	[42]
53	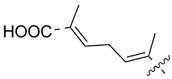	Isodomoic acid F	67	[42]
54	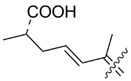	Isodomoic acid G		[41,42]
55	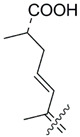	Isodomoic acid H		[41,42]

KARs, μM = displacement of kainic acid from the complex with kainate receptors.
